# The burden of CDI in the United States: a multifactorial challenge

**DOI:** 10.1186/s12879-023-08096-0

**Published:** 2023-03-07

**Authors:** Paul Feuerstadt, Nicolette Theriault, Glenn Tillotson

**Affiliations:** 1 Division of Digestive Disease, PACT-Gastroenterology Center, Yale University School of Medicine, Hamden, CT USA; 2 GSTMicro LLC, North, VA 23128 USA

**Keywords:** *Clostridioides difficile* infection, Recurrent *C. difficile* infections, Healthcare-associated, Community-acquired, Healthcare burden, Quality of life

## Abstract

*Clostridioides difficile* infection (CDI) affects approximately 500,000 patients annually in the United States, of these around 30,000 will die. CDI carries significant burdens including clinical, social and economic. While healthcare-associated CDI has declined in recent years, community-associated CDI is on the rise. Many patients are also impacted by recurrent *C. difficile* infections (rCDI); up to 35% of index CDI will recur and of these up to 60% will further recur with multiple recurrences observed. The range of outcomes adversely affected by rCDI is significant and current standard of care does not alter these recurrence rates due to the damaged gut microbiome and subsequent dysbiosis. The clinical landscape of CDI is changing, we discuss the impact of CDI, rCDI, and the wide range of financial, social, and clinical outcomes by which treatments should be evaluated.

## Background

**Fig. 1:**
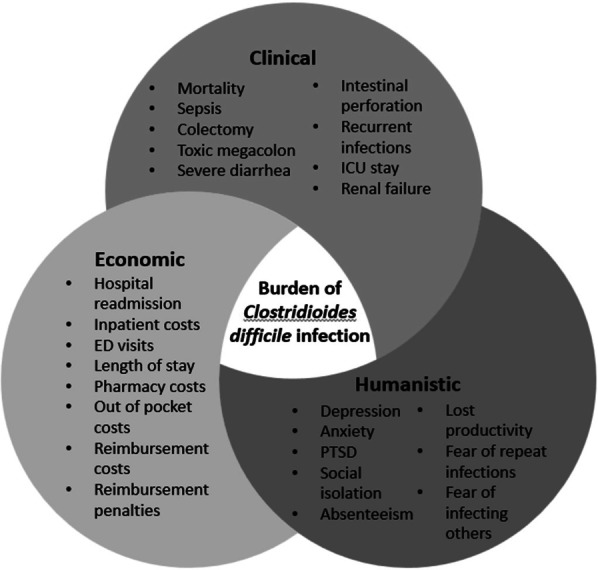
Burden of *Clostridioides difficile* infection.  *ED* emergency department; *ICU* intensive care unit; *PTSD* post-traumatic stress disorder

*Clostridioides difficile* infection (CDI) is the leading cause of antibiotic- and healthcare-associated infective diarrhea in the United States [1, 2]. The clinical presentation varies from asymptomatic colonization to mild diarrhea to severe debilitating disease, with high fever, severe abdominal pain, paralytic ileus, colonic dilation (or megacolon), or even perforation [3–5]. As such, the burden of CDI is high with substantial clinical, social, and economic implications (Fig. 1).

The Centers for Disease Control (CDC) identified CDI as an “urgent threat”, highlighting the need for immediate and aggressive action to prevent complications and recurrences of this infection [6]. The *C. difficile* surveillance program was launched in 2009 and six key components of prevention were stressed in the CDC’s 2012 *Vital Signs* report [7]. As a result, there is promising evidence that CDI rates have declined [8] but its epidemiology has shifted, and clinicians should be aware that it is no longer an infection that primarily affects patients in healthcare facilities. Additionally, there are a wide range of financial, social, and clinical outcomes which should be considered by clinicians when selecting treatment and patient care plans.

## The changing epidemiology of CDI

Despite some of the challenges of detecting *C. difficile*, the toxins produced, and the potential over-reporting based on PCR testing, CDI is associated with almost half a million infections and roughly 30,000 deaths annually in the US [1, 8, 9]. A recent meta-analysis estimates the CDI incidence to be 8.3 cases per 10,000 patient-days [10] and the CDC’s most recent surveillance data reports the crude overall incidence rate to be 121.2 cases per 100,000 persons [11]. Epidemiology among the pediatric population differs compared to those > 18 years old, this review discusses the burden of CDI in the adult population.

The burden of CDI was estimated to have decreased by 24% from 2011 to 2017 when correcting for changes in diagnostics between those years [8]. More recently, the impact of the COVID-19 pandemic on CDI prevalence has been a focus for several studies; while some have noted increased [12, 13] or stable prevalence/incidence [14, 15], most have demonstrated a decline [16–18] corresponding with decreased testing and extraordinary reinforcement of infection prevention measures [15, 18, 19]. Broader, more definitive data from the CDC would be helpful to best answer the impact the COVID-19 pandemic had on CDI incidence.

The CDC reports that the incidence rate of healthcare-associated CDI (defined as those with onset in a healthcare facility or associated with recent admission to a healthcare facility) is 57.9 cases per 100,000 persons [20], which represents a sizeable decline in recent years. While traditionally viewed as a nosocomial infection, data reveals that the incidence of healthcare-associated CDI cases in older patients was 47% in 2019, which is down from 53% to 2012 [21]. The total burden of healthcare-associated CDI also declined by 36% from 2011 to 2017 [8]. It is believed these declines can be attributed to better enforcement of institutional policies including aggressive treatment of CDI, more rigid antimicrobial stewardship and meticulous infection control practices.

Community-associated CDI, on the other hand, is on the rise, almost doubling in the past decade [22] with an incidence of 63.3 cases per 100,000 persons [20]. The incidence of community-associated CDI increased to 53% in 2019 compared to 47% in 2012 [21]. For reference, the CDC’s surveillance program classifies cases as community-associated if the *C. difficile*-positive stool specimen was collected on an outpatient basis or within 3 days after hospital admission in a person with no documented overnight stay in a healthcare facility in the preceding 12 weeks [20]. Given the changing epidemiology of CDI, continued efforts are required to improve infection prevention and diagnostic and antibiotic stewardship not only in inpatient settings, but also in outpatient settings.

## Recurrent CDI is on the rise

A major clinical challenge is recurrent CDI (rCDI); after an initial episode of CDI, between 20 and 35% of patients will experience a recurrence (usually within 30 days) [23–29]. Furthermore, of the patients who have a recurrence, up to 60% will experience subsequent recurrences [23, 25, 30, 31]. For example, Nelson et al. reported that 35% of the study population experienced rCDI; of those who experienced one recurrence, 59% had a second recurrence, and of those who had two recurrences, 58% had a third [23]. In fact, studies have shown that the risk of recurrence more than doubles after two or more recurrences [29].

The incidence of rCDI has increased significantly in recent years and this has been identified as a major public health challenge [1, 23, 32–34]. Data indicate that in the US, recurrence accounts for 75,000 to 175,000 additional cases of CDI per year [33].

Recurrences of CDI are believed to occur due to alterations of the colonic microbiota which shifts the protective metabolites, such as secondary bile acids and short chain fatty acids, and the replacement species such as Enterobacterales produce metabolites which lead to spore germination. During the time after antimicrobial treatment of CDI, without further intervention, the microbiota naturally replenishes its deficiencies but is frequently unable to successfully complete that process, resulting in a recurrence. In fact, the antimicrobials used to treat CDI, such as vancomycin, further deplete the microbiota leaving patients prone to further recurrence. Continued disruption of the normal colonic microflora by repeated cycles of antibiotic therapy used to treat rCDI perpetuates the risk of repeated recurrences [29]. This vicious cycle of infection–reinfection impedes recovery, thereby exacerbating the burden of CDI.

The breadth of outcomes reported to be associated with CDI is shown in Fig. 1.

## Risk factors for CDI and rCDI

The most important risk factor for CDI is antibiotic use, with 60% of CDI cases using antibiotics in the 4 months prior to infection [20, 35]. Ampicillin, amoxicillin, cephalosporins, clindamycin, and fluoroquinolones are the antibiotics most frequently associated with the infection, but almost all have been associated to some degree [35]. Antibiotics alter the normal gut flora, allowing *C. difficile* to proliferate in the gut where it produces three toxins: toxin A, toxin B, and occasionally binary toxin. Toxins A and B cause cytotoxicity and cellular detachment from intestinal epithelium, acting as the responsible agents for CDI symptomatology whereas binary toxin is thought to enhance toxin A and B toxicity [36, 37].

The risk for CDI and rCDI is higher among patients who are female, older, have comorbidities (including renal disease, liver disease, rheumatoid arthritis, multiple sclerosis, diabetes, and inflammatory bowel disease), are immunosuppressed, have recently been hospitalized, and have a history of using corticosteroids, proton pump inhibitors, or lipid-lowering therapy [1, 20, 26, 34, 38–44]. Greater age and the presence of multiple comorbidities are prognostic factors for severe CDI [44]. Not surprising, the elderly has been disproportionately affected and long-term care facilities have borne a significant proportion of the burden of CDI [45–48].

Patients diagnosed with community-acquired CDI differ somewhat in that they tend to be younger with a significant proportion reporting no antibiotic exposure during the 4 months prior to diagnosis [36, 41]. It remains unclear why this infection is now impacting a different demographic that has not received antimicrobials in the recent past.

## The clinical picture of CDI is heterogeneous

The clinical picture of CDI is heterogeneous, and ranges from an asymptomatic carrier state to a life-threatening colitis. Results of a recent meta-analysis reveals that *C. difficile* accounts for 20% of all antibiotic-associated diarrhea cases among hospitalized patients [49]. The extent of CDI can range from mild to profuse diarrhea, severe colitis, and rarely, toxic megacolon. That said, the majority of patients suffer from cramping abdominal pains with mild to moderate diarrhea and experience recovery within 3–5 days of antimicrobial initiation [36]. Associated signs and symptoms, seen in the more severe cases, may include nausea, vomiting, fever, abdominal pain, tenesmus, dehydration, abdominal distension, hypo-albuminemia with peripheral edema, and subsequent circulatory shock [36].

Asymptomatic carriage can be defined as the absence of diarrhea without colonoscopic or histopathologic findings consistent with pseudomembranous colitis, and either presence or detection of *C. difficile* toxins. The management of these patients depends on the setting and underlying conditions.

## CDI remains a significant threat for mortality

The clinical burden of CDI and rCDI is extensive. Hospitalized CDI patients are sicker, have higher severity illness scores, double the likelihood of loss of function, and double the likelihood of dying compared to patients hospitalized for all other diagnoses [50]. Studies from four different sources, CDC Emerging Infections Program (EIP) [11], Premier Healthcare Database [18], National Inpatient Sample [50] and Veterans Administration [51] reported 30-day CDI mortality rates ranging from 6 to 11% [1, 18, 51, 52], and all-cause data reveals that CDI patient mortality has increased throughout the COVID-19 pandemic [18]. Mortality rates associated with all-cause infections increase to 20–37% in the intensive care unit (ICU) setting [51–53] where patients have more than triple the odds of mortality compared to their non-ICU counterparts [54].

Mortality is also increased with rCDI [1, 28, 42]. CDI-associated deaths are almost ten times higher in older patients over the course of a year after rCDI (25.4%) than non-recurrent CDI (2.7%) [28]. Mortality rates also increase with number of recurrences, starting at 16% for those with one recurrence, 31% for those with two recurrences, and 39% for those with three or more recurrences [28]. Predictors of mortality in rCDI include use of proton pump inhibitors or antibiotics, respiratory failure, cognitive dysfunction, nutrition deficiency, age, and higher comorbidity scores [28, 52].

## The likelihood of complications is high with CDI and rCDI

Significant health complications of CDI include sepsis, colectomy, megacolon, intestinal perforation, and renal failure [42]. An analysis of three different sources, a commercial younger CDI related population, Medicare and all-cause data from the United Kingdom shows the incidence of complications and surgical intervention increases with rCDI, all of which contribute to longer hospital stays, ICU requirements, and high inpatient admission rates [38, 42, 55].

Due to disruption of the gastrointestinal epithelium by CDI toxins, translocation of bacteria can also predispose patients to bloodstream infections (BSI), including *Candida* spp., *Enterobacteriaceae*, and *Enterococcus* spp., which can have mortality rates as high as 60% [56–59]. Candidemia is particularly problematic as the mortality associated with candidemia is approximately 40%, but when the candidemia is subsequent to CDI, mortality reaches 57% [57].

**Fig. 2:**
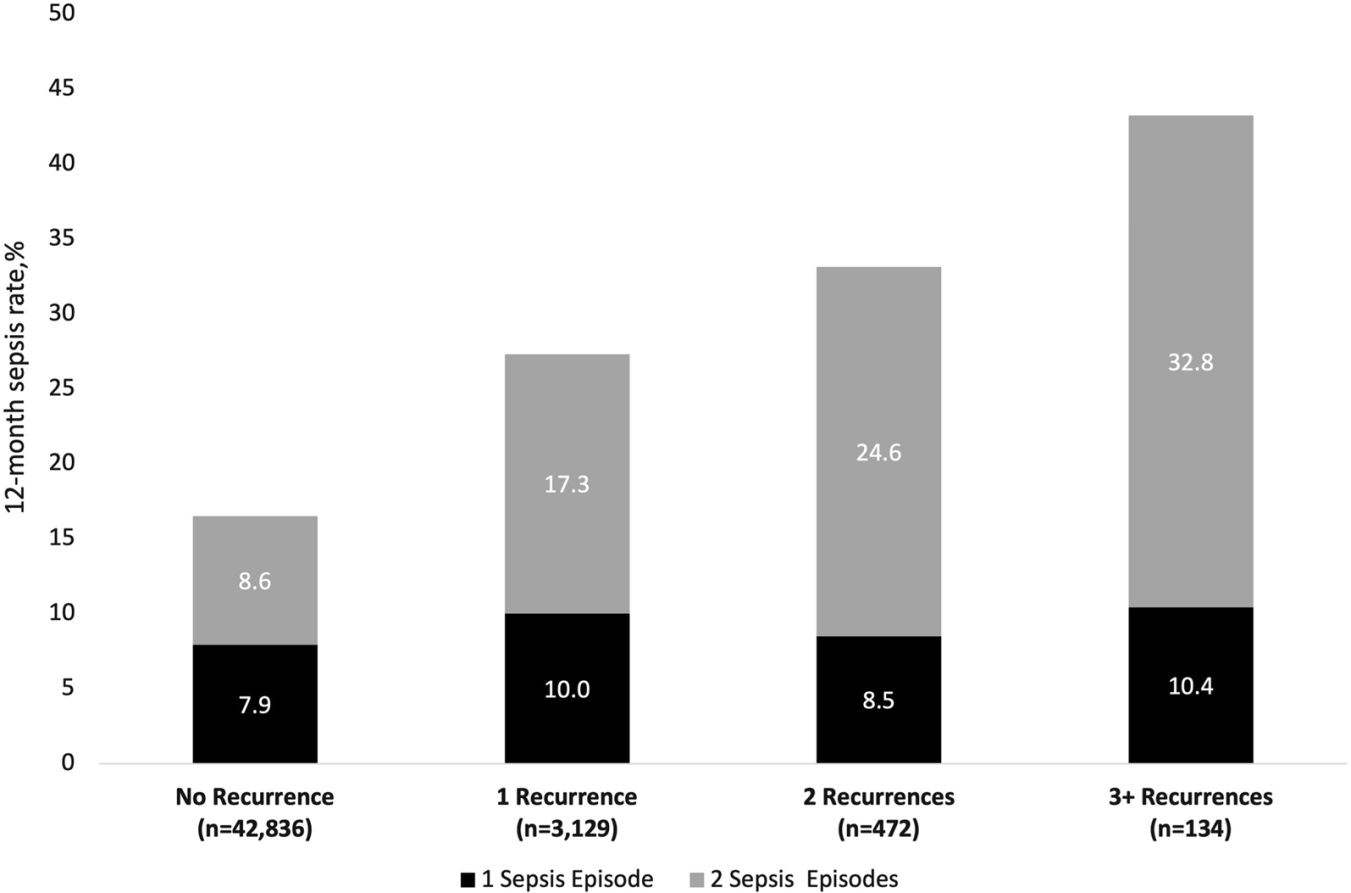
Rates of sepsis during the 12 months after an index CDI episode, by recurrence cohort. (Adapted from Feuerstadt et al. [38]; with permission)

Sepsis, defined as life-threatening organ failure caused by an infection, is a major cause of morbidity and mortality in CDI patients. The relationship between CDI and sepsis is complex. Assignment of the direct cause of one with the other is often difficult as the toxins produced have been shown to have a systemic impact while treatment with board-spectrum for infections such as sepsis can lead to the shift of the gut microbiome and emergence of *C. difficile*. The real-world study of CD-related CDI by Feuersdtadt et al. provides insight into the impacts of CDI upon both patients and the healthcare system. During a 12-month period, 16.5% of CDI patients experienced sepsis and this increased with recurrences (27.3% of patients with their first recurrence experienced sepsis, 33.1% with two recurrences, and 43.2% with three or more recurrences) (Fig. 2) [38]. Rates are higher for older patients with 39% of Medicare patients with CDI suffering sepsis, increasing to 45% in those with rCDI [55]. Mortality associated with sepsis is very high with in-hospital, 30-day, and 12-month mortality rates of 24% [60], 30% [61], and 58% [55], respectively.

**Fig. 3:**
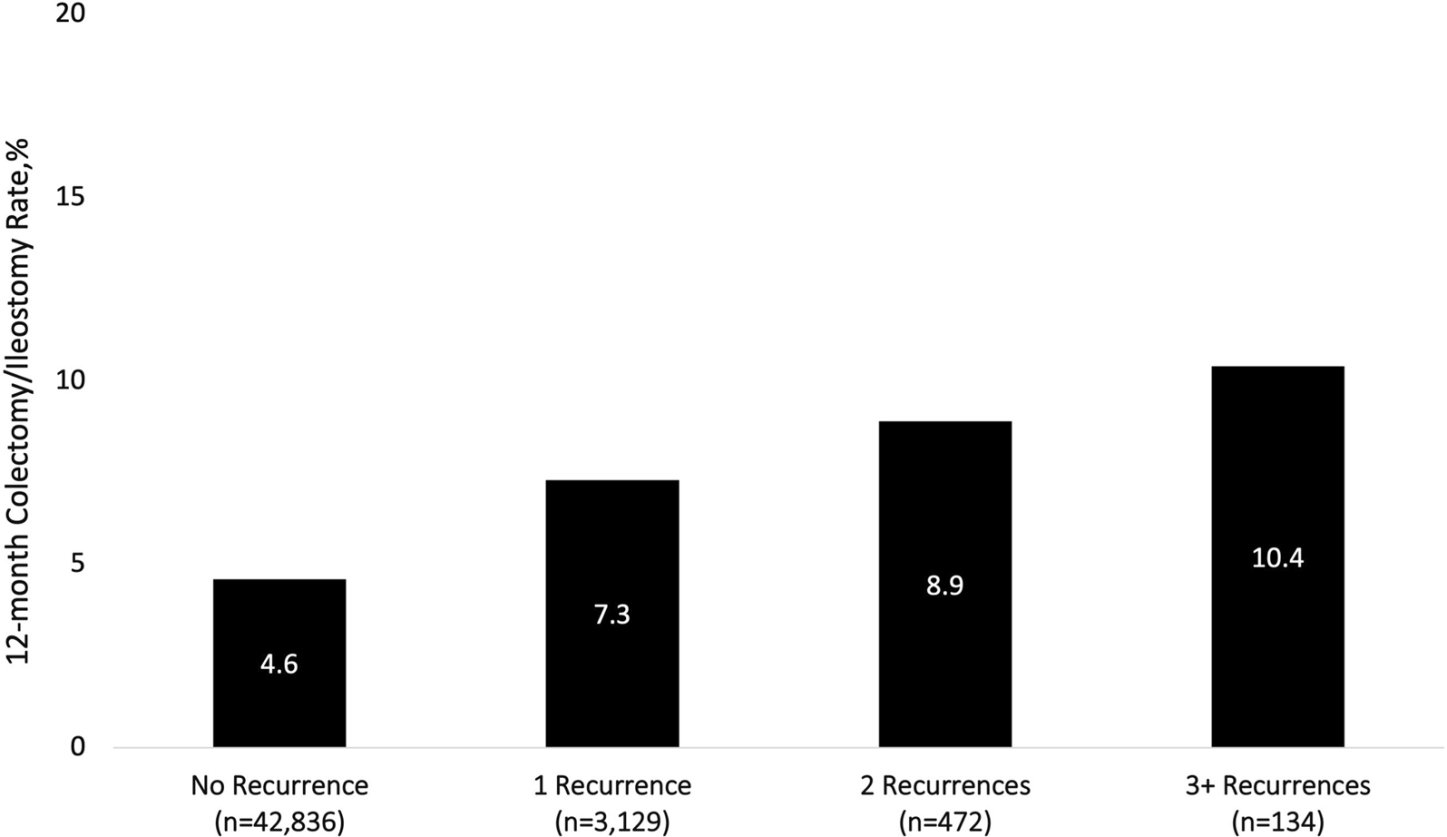
Rates of subtotal colectomy or diverting loop ileostomy during the 12 months after an index CDI episode, by recurrence cohort. (Adapted from Feuerstadt et al. [38]; with permission)

Almost 8% of patients hospitalized with CDI are afflicted with severe CDI or fulminant CDI and a significant proportion of these patients undergo colectomy or diverting loop ileostomy with colonic lavage [62]. Data reveal that over the course of a year, 4.6% of CDI patients undergo colectomies and this increases to 7.3% in those with one recurrence and > 10% in those with three or more recurrences (Fig. 3) [38]. While surgical management of CDI is potentially curative, it is by no means benign. In-hospital mortality rates following a procedure vary from 30 to 80% [62–69], while up to 75% and 78% of patients experience complications and serious morbidity, respectively, within 30 days of surgery [69].

## The impacts of CDI and rCDI on patient quality of life

Various studies have reported that CDI and rCDI have significant detrimental effects on patients’ quality of life that can have long-lasting and emotional impacts [70–76]. For many patients there are various psychological, social, professional, and economic impacts that should not be under-appreciated.

### Psychological and social

Lurienne et al. explored the consequences of CDI through the patients’ perspective in a cross-sectional study. Participants were grouped into those who had active disease (current CDI) and those who had a history of CDI (past CDI). Almost all current CDI respondents (94%) admitted their daily activities are impacted by the infection and 66% reported psychological consequences. The psychological impact is high with 92% of current CDI respondents reporting fear of worsening CDI and 80% worrying that certain foods might contribute to the worsening. The impact of CDI was also broad, affecting sleep patterns and respondents’ social lives (cited by 73.9% and 79.1% of CDI respondents, respectively). Consequences remain even after the infection clears with 57% of respondents noting that post-CDI symptoms remain and 41% believing they will never get rid of them [74].

Not surprising, rCDI patients also report significant quality of life implications with 42% reporting that they are very worried about getting sick again. Additionally, 31% are very worried about infecting others, 26% feel like prisoners in their own home/hospital, and 22% are unable or unwilling to eat. Some of these patients (22–32%) report eating out less, avoiding certain medications and public areas, and increasing probiotic use [77]. For older adults able to live at home, rCDI often has additional negative impacts with common complaints including loss of independence and inability to travel or enjoy normal activities due to fear of uncontrolled episodes of fecal incontinence or diarrhea [71, 78].

Finally, CDI has been found to be associated with mental health conditions, including depression and anxiety, which warrants further investigation [73, 79, 80].

### Professional

CDI has a major impact on patients’ professional lives. In the Lurienne et al. study, 74% of the current CDI respondents identified impacts on work activities with almost half (47%) reporting that they had to stop working while actively infected and another 26% responding that they had to stop working afterwards because of consequences from the episode. Once out of work, patients remain unable to perform their professional responsibilities for extended periods both during the infection (average of 118 days) and after clearance (average of 310 days) [74].

Another multinational study reported that CDI patients with active disease suffer diminished work productivity; the rate of productivity loss among CDI patients attending work (i.e., presenteeism) was nearly double that of those with no history of CDI whereas the rate of absenteeism was 2.5-fold higher [71].

### Economic

In addition to the need to take time off work, patients with CDI and rCDI are often left with substantial financial responsibilities. Patients report spending an average of $4355 out of pocket on current CDI treatment and $8695 on past CDI treatment [74]. Interestingly, total mean out-of-pocket cost is highest among community-associated hospitalized CDI patients versus hospital-associated or non-hospitalized CDI patients [21].

## The burden of CDI and rCDI on the healthcare system is high

CDI and rCDI are associated with a substantial economic burden that is driven by hospitalization costs (e.g., hospital admissions, intensive care use, length of stay) [21, 26, 28, 42, 55, 60, 81]. The number of all CDI-related hospitalizations has increased in recent years with CDI accounting for almost 1% of all admissions [50].

The cyclical nature of rCDI contributes to the significant burden with recurrence increasing the likelihood of hospitalization [23]. Rodrigues et al. reported that most patients (84%) with recurrence had a CDI-related hospitalization within 12 months [40] and another cohort study reported that 25% of CDI patients who survived an initial hospitalization were readmitted within 60 days [82]. Patients with CDI have an average hospital length of stay of 8 days for an index episode and 9.3 days for a rCDI episode [26]. Furthermore, patients with three or more recurrences have an average of 5.8 inpatient visits and 4.6 emergency department visits over the course of a year [81].

**Fig. 4:**
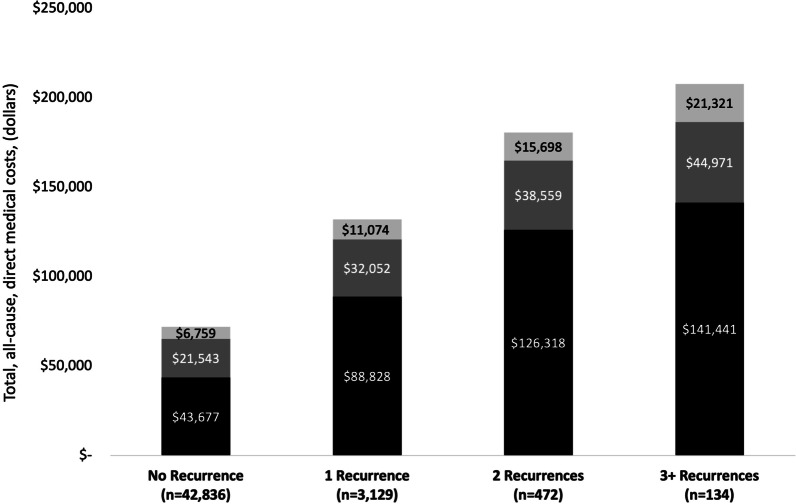
Total, all-cause, direct medical costs during the 12-month period after an index CDI episode, by recurrence cohort; costs adjusted to 2018 dollars. (Adapted from Feuerstadt et al. [81]; with permission)

Inpatient costs of CDI to the US are estimated to be nearly $5 billion annually [83] while rCDI costs are estimated to be $2.8 billion [40]. The average healthcare costs attributable to CDI over a 6-month follow up period are $39,000 with recurrence increasing costs up to $49,000 [26, 40]. The associated costs are certainly higher over a longer period (12 months) with mean total, all-cause, direct medical costs starting at $71,980, and ranging between $131,953 for patients with one recurrence to more than $200,000 in patients with three or more recurrences (Fig. 4) [81]. The COVID-19 pandemic increased the already high per-patient costs by roughly $2000 compared to pre-pandemic times [18].

Outpatient costs include outpatient hospital visits, physician office visits, emergency department visits, and other outpatient services such as laboratory and imaging tests [81]. While these costs are not inconsequential, it is the inpatient costs that are the key cost driver, accounting for almost 70% of the total CDI costs [40, 81], followed by surgery-related costs (20%) and treatment costs (8%) [40].

Hospital costs represent a fixed fraction of that which is billed but there are often revenue gaps that occur when payors provide the actual reimbursements. Hospital readmission with CDI as a primary diagnosis incurs a revenue loss of almost $5000 and the potential loss is nearly threefold higher for patients who are re-hospitalized with rCDI as a secondary diagnosis (nearly $14,000 per hospitalization) [82]. These results suggest that the healthcare system can benefit from more efficient models of care for these patients.

## Conclusion

CDI has many unseen and underappreciated consequences that go far beyond gastrointestinal symptomology. Clinical treatment and management need to be multifaceted and consider not just pharmaceutical intervention but a holistic approach to the patient’s experience both during and after CDI, acknowledging the potential psychological and social effects as well as identifying payment assistance programs, supportive services, and work medical leave options. From a health resource and healthcare institution perspective, efforts should be made to reduce costs and fiscal losses due to reimbursement penalties and efforts should be directed at preventing rCDI and community-acquired CDI.

## Data Availability

Not applicable.

## Abbreviations

rCDI: Recurrent *Clostridioides difficile* infections
CDI: *Clostridioides difficile* infection
CDC: Centers for Disease Control
ICU: Intensive care unit
BSI: Bloodstream infections
